# Novel insights into maintaining genomic integrity: Wee1 regulating Mus81/Eme1

**DOI:** 10.1186/1747-1028-6-21

**Published:** 2011-12-09

**Authors:** Yusé Martín, Raquel Domínguez-Kelly, Raimundo Freire

**Affiliations:** 1 Unidad de Investigación, Hospital Universitario de Canarias, Instituto de Tecnologías Biomédicas, Ofra s/n, La Laguna, Tenerife, Spain

**Keywords:** Wee1, Mus81/Eme1, Replication, Genomic integrity, Cell cycle, Cdk2

## Abstract

Maintenance of genomic integrity is essential for cell survival. Specifically, during DNA replication cells use a complex network of mechanisms that prevents genomic instability. Recently, we and others identified Wee1, a serine/threonine and tyrosine kinase, as a new modulator of the genomic stability during S phase. Loss of its activity causes a general DNA damage response activation and a decrease in replication fork speed. These effects are counteracted by the downregulation of the endonuclease complex Mus81-Eme1, showing a new link between this endonuclease and Wee1 during DNA replication. Here we discuss the function of Wee1 in genomic stability and its relationship with the Mus81-Eme1 complex.

## Introduction

Different external and internal agents can cause genomic alterations that trigger a variety of mechanisms allowing the cell to repair the injuries or avoid its cell cycle progression. The correct functioning of these pathways, generally called DNA Damage Response (DDR) is essential for maintaining genome integrity and for preventing the development of diseases associated with genomic instability [1].

The phosphatidylinositol 3-kinase like kinases (PIKKs) ATM and ATR are central kinases in the DDR. ATM responds to DNA double strand breaks (DSBs), while ATR is activated in response to a wider variety of DNA lesions such as UV-induced damage, replication stress and DSBs. After DNA damage, ATR and ATM, with the help of other DDR proteins, phosphorylate and thereby activate the two effector kinases Chk1 and Chk2, respectively, which are essential to transmit the upstream signal to the downstream proteins [2].

DNA replication can be a risky process and when replication is compromised the DNA is more vulnerable to suffer breakages [3]. Under these conditions, DNA secondary structures such as stalled replication forks are formed, which triggers a DDR that stabilizes the fork thereby avoiding replication fork collapse. Besides DDR proteins, specific enzymatic activities are required, for example the Mus81/Eme1 dimer, to revert stalled replication forks. This heterodimeric complex is a structure-specific endonuclease, where Mus81 is the catalytic subunit and Eme1 the regulatory factor [4]. This enzyme is involved in the cleavage of stalled and blocked replication fork by introducing DSBs to allow the replication recovery [5,6].

Wee1 is a serine/threonine and tyrosine kinase that was first identified as a Cyclin-dependent kinase (Cdk) regulator [7,8]. Wee1 protein levels are highly regulated during the cell cycle. Wee1 activity and levels are high during S and G2 phases, when Wee1 is able to inhibit Cdks by phosphorylating them at tyrosine 15 and threonine 14 [9]. During the G2/M transition, Wee1 is phosphorylated by Plk1, which triggers its degradation by proteasome and allows mitotic entry [10].

Recently, we and others described a novel function for Wee1 in maintaining genomic integrity, by ensuring proper DNA replication [11,12]. Our results indicate that Wee1 is involved in regulating the Mus81-Eme1 endonuclease activity to avoid undesirable DNA breaks.

## Wee1 activity controlling genomic stability

Wee1 is a well-known negative regulator of Cdks and hence a controller of cell cycle progression. Only recently, Wee1 has emerged as a key protein for maintaining genomic integrity both in human and in plant cells and its kinase activity seems to be necessary for proper DNA replication in unperturbed cells [11-14]. Downregulation of Wee1 or its chemical inactivation causes a general activation of the DDR with a large accumulation of phosphorylated histone H2AX, a hallmark of the DDR, in S phase cells. Along with an activation of DDR, a slow-down in replication fork speed is observed. These data demonstrate a novel role for Wee1 beyond its known role in the G2/M transition.

Like Wee1, Chk1 is also active during a normal cell cycle having an important role in ensuring the correct replication of the DNA during S phase [15,16]. Similar to the effects of Wee1 depletion, inhibition or downregulation of Chk1 causes destabilization of the genome, accumulation of large amounts of DNA breaks and slower replication fork speed [15,16]. In Xenopus, the function of both kinases was described to be closely related, and activated Chk1 phosphorylates Wee1 thereby promoting its Cdk-inhibiting activity [17]. A possible explanation for the phenotypic similarities of the lack of both proteins during S phase could therefore be that they act in the same pathway. Chk1 might regulate Wee1 activity and consequently, controlling genomic integrity during S phase. However, our results discard this option as we observed that inhibition of both kinases show additive effects. The inhibition of both Wee1 and Chk1 causes a further decline in the replication fork speed and cells transfected with Wee1 and Chk1 siRNA oligos are less viable with a clear increase in cell death comparing to single downregulated cells [11]. Consequently, both kinases are important in maintenance of genomic integrity during S phase but act at different points. A recent work shows that enzymatic inhibition of both kinases simultaneously has a synergistic effect inhibiting cell proliferation [18], which confirms our findings.

Our work demonstrates that the DDR activation upon Wee1 inhibition critically depends on the endonuclease complex Mus81/Eme1. In fission yeast, Mus81/Eme1 cellular localization is regulated by Cds1 (functional ortholog of human Chk1) and plays a role in S phase [19]. After hydroxyurea treatment, Cds1 phosphorylation releases Mus81/Eme1 from the chromatin, thereby preventing cleavage of the replication fork. In humans this relationship remains unclear although it was recently shown that Mus81 depletion in Chk1 (funtional ortholog of Cds1 in human cells)-inhibited cells reduces damage accumulation and partially increases the replication fork speed [20].

**Figure 1:**
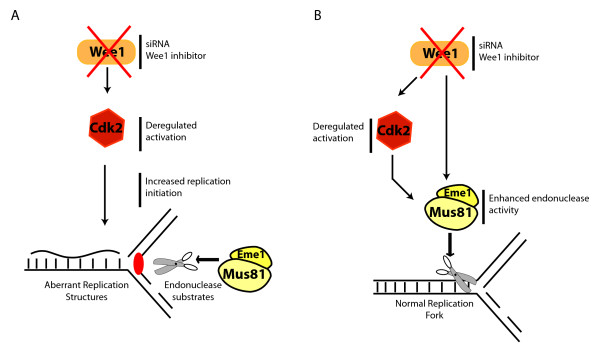
**Link between Wee1 and Mus81**. **A**. Lack of Wee1 hiperactivates Cdk2 that deregulates DNA replication and produces DNA aberrant structures that are substrate of Mus81/Eme1. **B**. Wee1 kinase regulates the Mus81/Eme1 endonuclease complex, either indirectly through inhibition of cyclin-dependent kinase Cdk2, which could be an activator of Mus81, or directly through phosphorylation and inhibition of Mus81/Eme1 complex.

How does Wee1 inhibition induce a DDR? We consider two possibilities to explain the Mus81-dependent DDR in the absence of Wee1: inhibition of Wee1 promotes deregulation of replication initiation, thereby creating substrates for the Mus81 enzyme (Figure 1A). Alternatively, Wee1, could regulate, directly or indirectly, Mus81 activity during S phase (Figure 1B).

Regarding the first hypothesis: It is known that the initiation of DNA replication requires Cdk kinases and among them, Cdk2 is considered the key kinase [21,22], although Cdk1 can compensate its absence [23]. Cdk2 activity precisely controls origins firing, regulating the recruitment of initiation of replicating proteins CDC45L and CTF4 to these sites [24,25]. To prevent an excessive number of replication origins, Cdk2 must therefore be tightly regulated. Hyperactivation of Cdk2 by deletion of Wee1 could cause a loss of control of the replication coordination that can result in aberrant DNA structures cleaved by Mus81 (Figure 1A). Althought we demonstrated that Cdk2 is phosphorylated by Wee1 during S phase, we did not observe major changes in chromatin loading of replication proteins upon Wee1 depletion and we observed a quick DDR activation upon enzymatic inhibition of Wee1 in cells synchronized in S phase. These and other data make us favour a regulation of Mus81 by Wee1 during S phase, as proposed in the second hypothesis.

In line with our results, Matos and collaborators have recently shown that a tight regulation of Mus81/Eme1 during mitosis and meiosis exists in both human and budding yeast cells [26]. This regulation depends on Plk1 kinase or its ortholog in yeast, Cdc5, that phosphorylates Eme1/Mms4 component allowing Mus81 activation which is required to resolve holliday junctions that bind sister chromatids [26].

Our observations show that the DDR caused by Wee1 inactivation is counteracted both by the depletion of Cdk2 and the depletion of Mus81. One possible explanation for these results is that Cdk2 regulates Mus81/Eme1 activity or localization. In this scenario Wee1 might inactivate Cdk2 in an unperturbed S phase thereby keeping Mus81/Eme1 inactive, likely as a consequence of its non-phosphorylation by Cdk2. In the absence of Wee1, Cdk2 might overactivate Mus81/Eme1 leading to the cleavage of unwanted substrates such as replication forks, which would slow down replication progression and increase genomic instability (Figure 1B). Nevertheless, our results favour a direct role for Wee1 in controlling Mus81, as we are able to detect an interaction between these two proteins. Therefore, we suggest that during a normal S phase Wee1 inhibits the Mus81/Eme1 complex directly either by inhibiting its activity *per se *or altering the substrate recognition, as we could not detect any change in chromatin loading of Mus81 in Wee1 depleted cells [11]. In the absence of Wee1 activity, Mus81 is activated and recognises and cleaves different substrates (Figure 1B). Linking our results with the recent work of the Plk1-dependent mitotic activation of Mus81, it is known that Plk1 regulates Wee1 by sending it for degradation during mitosis. This suggests the presence of a Mus81 regulation pathway during the cell cycle involving these two kinases. Depletion of Wee1 would allow the endonuclease activity of Mus81/Eme1 to successfully complete a correct chromosome segregation required at this phase of the cycle.

However, at the moment we cannot rule out any of these hypotheses and further experiments are required to identify the direct regulation of Mus81 by Cdk2 and/or Wee1 kinases.

## Conclusion

We and others identified Wee1 as a new modulator of genomic stability and its activity is essential for correct DNA replication, preventing an increase of genomic breaks created by Mus81/Eme1 activity. We showed an interaction between Wee1 and Mus81 and suggest a model in which Wee1 directly regulates Mus81 activity during S phase, possibly by phosphorylation. Finally, since the lack of Wee1 induces damage specifically in replicating cells, these recent discoveries made Wee1 a promising antitumoral-target.

## Competing interests

The authors declare that they have no competing interests.

## Authors' contributions

YM, RDK and RF wrote the manuscript. The authors read and approved the final manuscript.
